# Immunomodulatory Effects of Dietary Seaweeds in LPS Challenged Atlantic Salmon *Salmo salar* as Determined by Deep RNA Sequencing of the Head Kidney Transcriptome

**DOI:** 10.3389/fphys.2018.00625

**Published:** 2018-06-01

**Authors:** Arjan P. Palstra, Jeroen Kals, Ainhoa Blanco Garcia, Ron P. Dirks, Marnix Poelman

**Affiliations:** ^1^Wageningen Marine Research, Wageningen University & Research, Yerseke, Netherlands; ^2^Department of Animal Breeding and Genomics, Wageningen Livestock Research, Wageningen University & Research, Wageningen, Netherlands; ^3^Department of Animal Nutrition, Wageningen Livestock Research, Wageningen University & Research, Wageningen, Netherlands; ^4^ZF-Screens B.V., Leiden, Netherlands

**Keywords:** Atlantic salmon *Salmo salar*, aquaculture, dietary seaweeds, immune response, RNAseq, head kidney transcriptome

## Abstract

Seaweeds may represent immuno-stimulants that could be used as health-promoting fish feed components. This study was performed to gain insights into the immunomodulatory effects of dietary seaweeds in Atlantic salmon. Specifically tested were 10% inclusion levels of *Laminaria digitata* (SW1) and a commercial blend of seaweeds (Oceanfeed^®^) (SW2) against a fishmeal based control diet (FMC). Differences between groups were assessed in growth, feed conversion ratio and blood parameters hematocrit and hemoglobin. After a LPS challenge of fish representing each of the three groups, RNAseq was performed on the head kidney as major immune organ to determine transcriptomic differences in response to the immune activation. Atlantic salmon fed with dietary seaweeds did not show major differences in performance in comparison with fishmeal fed fish. RNAseq resulted in ∼154 million reads which were mapped against a NCBI *Salmo salar* reference and against a *de novo* assembled *S. salar* reference for analyses of expression of immune genes and ontology of immune processes among the 87,600 cDNA contigs. The dietary seaweeds provoked a more efficient immune response which involved more efficient identification of the infection site, and processing and presentation of antigens. More specifically, chemotaxis and the chemokine-mediated signaling were improved and therewith the defense response to Gram-positive bacterium reduced. Specific *Laminaria digitata* effects included reduction of the interferon-gamma-mediated signaling. Highly upregulated and specific for this diet was the expression of *major histocompatibility complex class I-related gene protein*. The commercial blend of seaweeds caused more differential expression than *Laminaria digitata* and improved immune processes such as *receptor-mediated endocytosis* and *cell adhesion*, and increased the expression of genes involved in *response to lipopolysaccharide* and *inflammatory response*. Particularly, expression of many important immune receptors was up-regulated illustrating increased responsiveness. *NF-kappa-B inhibitor alpha* is an important gene that marked the difference between both seaweed diets as *Laminaria digitata* inhibits the expression for this cytokine while the blend of seaweeds stimulates it. It can be concluded that the inclusion of seaweeds such as *Laminaria digitata* can have important modulatory effects on the immune capacity of Atlantic salmon resulting in a more efficient immune response.

## Introduction

Seaweed additives in fish feed may have beneficial physiological effects without affecting growth performance negatively. Seaweeds may represent immuno-stimulants with anti-bacterial activity that could be used as health-promoting fish feed components thereby offering an alternative for the use of antibiotics (Bansemir et al., 2006). Although the total lipid content is generally low, seaweeds represent a good source of health promoting PUFAs as compared to other feed ingredients derived from plant and animal sources (Rajapakse and Kim, 2011). The increased immune capacity and improved disease resistance of fish by applying dietary seaweeds has been reviewed by Reverter et al. (2014) and reported by many papers ever since (e.g., Peixoto et al., 2016; Valente et al., 2016). Specifically, when we consider *Laminaria digitata* effects, several papers report on the beneficial immune effects of dietary Ergosan, which is based on *Laminaria digitata* and *Ascophyllum nodosum* extracts. These effects include the initial elevation in serum lysozyme and complement activity in seabass (*Dicentrarchus labrax*; Bagni et al., 2005) and the enhanced mucosal immune response in rainbow trout (*Oncorhynchus mykiss*; Sheikhzadeh et al., 2012). Oceanfeed^®^ effects include the increase of higher total fatty acid and LC n-3 PUFA concentrations in the flesh of farm raised Atlantic salmon (Wilke et al., 2015), but specific immune effects have not yet been reported.

Immunomodulatory effects are generally studied by subjecting experimental groups to injection with an immune system activator such as lipopolysaccharide (LPS) which mimics a bacterial infection (MacKenzie et al., 2004, 2008). In contrast to humans, fish are often resistant to the endotoxic shock caused by LPS (review by Swain et al., 2008). LPS is even applied in aquaculture as an immuno-stimulative tool to promote disease and stress resistance. LPS effects in salmonids may include the polyclonal proliferation of lymphocytes, respiratory burst, phagocytic activity of macrophages, effect modification of other immune agents, increased cytokine expression and interferon induction (reviewed by Salinas et al., 2004). The endotoxic shock is absent because LPS fails to induce antiviral genes downstream of the Toll-like receptor 4 (Iliev et al., 2005).

High-throughput transcriptome sequencing (RNAseq) represents an efficacious first-step methodology to map the most important pathways in a physiological response (Palstra et al., 2013; Rurangwa et al., 2015) including the impact of nutrition on the immune system of fish (Martin and Król, 2017). Transcript mapping, expression profiling and gene ontology of immune genes in the head kidney as the most involved organ in the innate and adaptive immune response of fish (Kaattari and Irwin, 1985) should reveal the major pathways and any significant immunomodulation by experimental factors.

This study was performed to gain insights into the immunomodulatory effects of dietary seaweeds in Atlantic salmon. Specifically tested were 10% inclusion levels of *Laminaria digitata* (SW1) and a commercial blend of seaweeds (Oceanfeed^®^) (SW2) against a fishmeal based control diet (FMC). Differences between groups were assessed in growth, feed conversion ratio (FCR) and blood parameters hematocrit (Hct) and hemoglobin (Hb). After a LPS challenge in fish representing each of the three groups, RNAseq was performed on head kidney tissue of individual fish to determine transcriptomic differences in response to the immune activation, to our knowledge for the first time in fish in this context.

## Materials and Methods

### Ethics Statement

The experimental protocols complied with the current laws of the Netherlands and were approved by the animal experimental committee (DEC nr. 2013113).

### Experimental Diets

*Laminaria digitata* was made available by North Seaweed (Kapelle, Netherlands) and Oceanfeed was purchased from Ocean Harvest Technology (Milltown, Ireland). Two experimental diets were tested: a diet with 10% *Laminaria digitata* (SW1) and a diet with 10% of a commercial blend of seaweeds (Oceanfeed^®^) against a control diet based on fishmeal (FMC). In this study, seaweed is tested as an organic supplement for fish feed which, following EFSA, requires a control group that does not include the additive and a treatment group dosed at use-level with the additive. *Laminaria* consisted of crude protein 11.1%, ether extract 1.1%, crude fiber 5.6%, and ash 36.4%. For Oceanfeed these values were: crude protein 10.9%, ether extract 0.98%, crude fiber 8.8%, and ash 49.3%. The diets were prepared using extrusion in cooperation with Research Diet Services (RDS, Wijk bij Duurstede, Netherlands). Diets FMC, SW1 and SW2 were isonitrogenous, isoenergetic, equal in amino acid composition, calcium and phosphates levels (**Tables 1–3**).

**Table 1:** Experimental set-up and recipes.

Recipes	Dietary treatment		
	FMC	SW1	SW2
**Basal ingredients (%)**			
Fishmeal^a^	30.600	30.600	30.600
Wheat gluten^b^	15.058	15.058	15.058
Wheat flour^c^	17.368	13.500	13.500
Fishoil^d^	13.500	13.319	13.319
Monocalcium phosphate^e^	3.071	3.071	3.071
Sonac hemoglobin 92P^f^	2.841	2.841	2.841
CPSP-G^g^	2.700	2.700	2.700
Amino acid mix salmon^1^	2.536	2.536	2.536
Premix salmon^2^	2.025	2.025	2.025
Linseed oil^h^	1.629	1.629	1.629
Soya lecithine^i^	0.999	0.999	0.999
Lime^j^	0.901	0.901	0.901
Soya oil^k^	0.470	0.470	0.470
Cholesterol^l^	0.297	0.297	0.297
Carophyll Pink^m^	0.054	0.054	0.054
	**90%**	**90%**	**90%**
**Test ingredients (%)**		
Wheat gluten^b^	0.212	0.000	0.000
Wheat flour^c^	3.868	0.000	0.000
CMC^n^	0.690	0.000	0.000
Diamol^m^	4.473	0.000	0.000
Soy protein concentrate^o^	0.515	0.000	0.000
Corn gluten meal^p^	0.242	0.000	0.000
Semolina^q^	0.001	0.000	0.000
Laminaria digitata^r^	0.000	10.000	0.000
Ocean feed^s^	0	0.000	10.000
	**10%**	**10%**	**10%**
Check	100	100	100

*^*a*^Danish LT fishmeal. Type LT (Triple Nine Fish Protein Esbjerg, Denmark), ^*b*^Wheat gluten, Gluvital 21000 Cargill, Netherlands, ^*c*^Wheat flour, ^*c*^Wheatflour (Meneba Weert, Netherlands), ^*d*^Coppens International, Netherlands, ^*e*^Tessenderlo Chemie, Belgium, ^*f*^Hemoglobin powder (Sonac, Bad Bramstedt, D), ^*g*^Sopropeche Boulogne sur Mer, ^*h*^Linagro, Lichtervelde, Belgium, ^*i*^Nutripur G Cargill, Hamburg, Germany, ^*j*^Inducal 250 van Sibelco/Ankerpoort, Maastricht, ^*k*^Soya oil refined, (Smilde foods, Heerenveen), ^*l*^Cholesterol SF van Dishman Netherlands B.V., Veenendaal, ^*m*^CAROPHYLL PINK, F. Hoffmann-La Roche Ltd., ^*n*^Carboxy methyl cellulose, ^*f*^Damolin A/S, Hamburg, ^0^Soycomil ADM Eurpoort B.V., Netherlands, ^*p*^Cargill, Sas van Gent, Netherlands, ^*q*^Fa Gebr van Eck, Wijk bij Duurstede, Netherlands, ^*r*^North SeaWeed, Netherlands, ^*s*^Ocean Harvest, Ireland. ^*1*^Amino acid mix: lysine 5 g kg^-*1*^, threonine 2 g kg^-*1*^, methionine 3 g kg^-*1*^, arginine 5 g kg^-*1*^, histidine 5 g.kg^-*1*^. ^*2*^Premix: vitamins (mg or IU kg^-*1*^ diet): vitamin A (retinyl acetate), 15000 IU; D3 (cholecalciferol), 3000 IU; K3 (menadione), 8 mg; B_*12*_ (cyanocobalamin), 0.10 mg; B1 (thiamine), 10 mg; B2 (riboflavin), 15 mg; B6 (pyridoxine hydrochloride), 15 mg; folic acid, 10 mg; biotin, 0.5 mg; inositol, 600 mg; niacin, 50 mg; pantothenic acid, 50 mg, choline chloride, 2000 mg; vitamin C, 300 mg; vitamin E, 500 mg; minerals (g or mg kg^-*1*^ diet): Mn (manganese sulfate), 30 mg; I (potassium iodide), 5 mg; Cu (copper sulfate), 5 mg; Co (cobalt sulfate), 2 mg; Cr (chromium sulfate), 1 mg; Mg (magnesium sulfate), 300 mg; K (potassium chloride), 2600 mg; Zn (zinc sulfate), 100 mg; Se (sodium selenite), 1 mg; Fe (iron sulfate), 60 mg. BHT (E300-321), 100 mg; calcium propionate, 1000 mg.*

**Table 2:** Calculated and analyzed proximate composition of diets.

Code	Unit	FMC	SW1	SW2
**Calculated (macro) nutritional composition**		
DM	(g.kg^-1^)	911	912	909
Ash	(g.kg^-1^)	128	121	134
CP	(g.kg^-1^)	434	435	434
EE	(g.kg^-1^)	213	213	212
CF	(g.kg^-1^)	8.5	6.9	10.1
NFE	(g.kg^-1^)	126	136	117
P	(g.kg^-1^)	14.1	14.0	14.0
Ca	(g.kg^-1^)	16.6	16.6	16.6
GE	(MJ.kg^-1^)	21.1	21.2	20.9
CP/GE	–	20.6	20.5	20.8
DM	(g.kg^-1^)	926	947	965
Ash	(g.kg^-1^)	136	126	139
CP	(g.kg^-1^)	469	467	469
EE	(g.kg^-1^)	134	126	118
CF	(g.kg^-1^)	3	6	6
NFE	(g.kg^-1^)	184	223	232
GE	(MJ.kg^-1^)	16.7	16.4	16.2
CP/GE	–	28.0	28.4	29.0

**Table 3:** Calculated amino acid composition.

Code	Unit	FMC	SW1	SW2
**Calculated amino acids**		
Lysine^a^	g.kg^-1^	29.4	29.7	29.3
Methionine^a^	g.kg^-1^	12.8	12.8	12.7
Cysteine^b^	g.kg^-1^	5.4	5.3	5.4
Threonine^a^	g.kg^-1^	16.9	17.1	17.0
Tryptophan^a^	g.kg^-1^	4.3	4.2	4.3
Isoleucine^a^	g.kg^-1^	15.3	15.5	15.3
Arginine^a^	g.kg^-1^	27.0	32.9	32.9
Phenylalanine^a^	g.kg^-1^	28.4	27.0	26.9
Histidine^a^	g.kg^-1^	17.4	17.4	17.2
Leucine^a^	g.kg^-1^	30.8	30.9	30.5
Tyrosine^b^	g.kg^-1^	12.5	12.7	12.5
Valine^a^	g.kg^-1^	19.8	20.1	19.8
Alanine	g.kg^-1^	21.3	22.2	24.9
Asparagine	g.kg^-1^	30.6	30.9	30.8
Glutamate	g.kg^-1^	80.4	79.0	80.4
Glycine	g.kg^-1^	22.4	22.6	22.4
Proline	g.kg^-1^	28.3	28.0	27.9
Serine	g.kg^-1^	17.6	17.5	17.6

*^*a*^Essential, ^*b*^conditionally essential.*

### Experimental Animals and Procedures

Atlantic salmon (378 ± 57 g) were purchased as juveniles from Meridian Salmon (Furnace, United Kingdom) and transported by Solway (Dumfriesshire, United Kingdom) to the experimental aquaculture facilities of Wageningen Marine Research (WMR) in Yerseke (Netherlands). Fish were accommodated in nine tanks (800 l each) which were integrated in a single recirculation system. The experiment consisted of a 15-day acclimatization followed by a 42-day experimental period. Diets were tested in triplicate with 20 fish per tank, adding up to 180 fish in total.

Fish were fed using feeding belts and feeding level was restricted. Tanks were checked frequently to verify if all feed was consumed. Dry matter content of diets was analyzed at the start of the experiment to be able to determine equal feeding levels on dry matter for all diets. Proximate compositions were analyzed by Nutrilab B.V. (Rijswijk, Netherlands).

Husbandry conditions during the experimental period were: photoperiod 16L:8D, temperature 13.8 ± 1.6°C, oxygen 8.0 ± 0.5 mg.l^-1^, pH 7.51 ± 0.04, TAN (total ammonia nitrogen) 0.05 ± 0.09 mg.l^-1^, NO_2_^-^, 0.07 ± 0.05 mg.l^-1^, salinity 25–30 ppt and flow 39.5 ± 2.3 l.min^-1^, which stayed within pre-set limits. Temperature and oxygen were measured daily. Flow, pH, TAN, NO_2_^-^ were measured weekly.

At the start and at the end of the experimental period all fish per tank (*N* = 20) were anesthetized using phenoxy ethanol (2 ml l^-1^) and weighed. Fish were not fed 1 day prior to weighing. *N* = 18 fish at the start and *N* = 10 fish per tank at the end of the experiment were sampled for blood to determine Hct and Hb. Blood was obtained by caudal venous puncture, using a heparinized syringe (0.6 mm/60 mm needle). The samples were transferred to Eppendorf tubes, stored on ice and processed within 15 min. Hct was determined by centrifuging blood samples for 5 min at 11,000 rpm (SpinCrit micro hematocrit centrifuge, Indianapolis, IN, United States). Hb content was determined using the method described by van Kampen and Zijlstra (1961).

At the end, *N* = 1 fish per tank (or *N* = 3 fish per diet) were subjected to a LPS challenge (LPSs from *Escherichia coli*; 6 mg.kg^-1^ in 0.2 ml physiological salt solution injected intraperitoneally) and between 68 and 70 h after injection, fish were sacrificed and dissected for head kidney tissue that was stored in RNAlater (Ambion). Sham injections were not applied as their effects would be filtered out by the comparisons that were made in our experimental design, e.g., comparing each of the experimental groups vs. the control. The potential effect of the intraperitoneal injection itself was not expected to be any different between the individuals of the three experimental groups, and certainly not for sequencing RNA expression in the head kidney.

### Growth

From the individual weight data, average body weight at the start (BW_0_) and at the end (BW_t_) was calculated per tank as experimental unit. Feed intake (FI) and FCR were calculated as well as growth rates in %.day^-1^ and g.kg^-0.8^.day^-1^. The specific growth rate (SGR) and growth expressed in metabolic body weight (GMBW) during the growth period were calculated with the formulas:

(1) $$\begin{array}{c} \mathrm{SGR}\;=\;(\mathrm{LN}({\mathrm{BW}}_{t})\;–\;\mathrm{LN}({\mathrm{BW}}_{0}))/(t)*100, \end{array}$$

with t as the duration of the growth period and LN the natural logarithm,

and

(2) $$\begin{array}{c} \mathrm{GMBW}\;=\;({\mathrm{BW}}_{t}\;–\;{\mathrm{BW}}_{0})/({\left({BWt*BW}_{0}\right)}^{0.5}{/1000}^{0.8})/t \end{array}$$

### Statistics of Growth and Blood Data

Data were analyzed using ANOVA to test for diet effects. Homogeneity of variance was tested using Levene’s test. When necessary, data were transformed or tested using the Kruskal–Wallis test. For all tests a probability *p* < 0.05 was considered significant. When significant, depending on the hypothesis; equal, higher- or lower, mean values were compared using the one sided or two sided Fisher’s LSD multiple comparisons *post hoc* test.

### RNAseq

RNA was isolated from homogenized head kidney samples (TissueRuptor, Qiagen, Venlo, Netherlands) of the three individual fish per diet using the miRNAeasy mini kit (Qiagen). RNA concentrations were between 158 and 1,200 ng μl^-1^ and RIN values ∼10. Illumina multiplexed RNAseq libraries were prepared from 2 μg total RNA using the Illumina TruSeq RNA Sample Prep Kit v2 according to the manufacturer’s instructions (Illumina Inc.). All nine RNAseq libraries (3 individual fish × 3 diets = 9 samples) were sequenced on an Illumina HiSeq 2500 sequencer as 1 × 50 nucleotides paired-end (PE50) reads according to the manufacturer’s protocol. Image analysis and base calling were done by the Illumina pipeline. A total of ∼154 million single-read 1 × 50 nt reads (∼8 Gb data) were derived from all nine RNAseq libraries. Two strategies were used for quantitative analysis of the head kidney RNAseq data sets: referred to as mapping reads against NCBI *Salmo salar* reference (strategy 1) and mapping reads against *de novo* assembled *Salmo salar* reference (strategy 2). Strategy 1 was more appropriate for salmon and particularly performed for analyses on gene level while strategy 2 was performed for more extensive and comprehensive data analyses on biological process level and detection of new immune salmon genes.

### Mapping Reads Against NCBI *Salmo salar* Reference

In strategy 1, reads were aligned to 48,223 *Salmo salar* cDNA sequences (downloaded from NCBI) using TopHat (version 2.0.5) (Trapnell et al., 2009) and about 15% of the RNAseq reads could be mapped. Reference alignment was done and the resulting files were filtered using SAMtools (version 0.1.18) (Li et al., 2009) to exclude secondary alignment of reads. For statistical comparison of gene expression levels between groups, aligned fragments per predicted gene were counted from SAM alignment files using the Python package HTSeq (version 0.5.3p9) (Anders et al., 2014). In order to make comparisons across samples possible, these fragment counts were corrected for the total amount of sequencing performed for each sample. As a correction scaling factor, we employed library size estimates determined using the R/Bioconductor (release 2.11) package DESeq (Anders and Huber, 2010). Read counts were normalized by dividing the raw counts obtained from HTSeq by its scale factor. Correction for false positives is included in the statistical analysis of gene expression through DESeq.

### Mapping Reads Against *de Novo* Assembled *Salmo salar* Reference

In strategy 2, CLC bio’s *de novo* assembler was used to generate cDNA contigs from the total of ∼154 million reads. This resulted in 87,600 cDNA contigs corresponding to mRNAs that were expressed in the head kidneys and ranging in size from 200 to 14,086 nt. The *de novo* cDNA contigs were annotated to known genes via custom BLAST searches against four reference databases: (a) a UNIPROT protein database (36.3% hits), (b) a Teleost Refseq mRNA database (26.5% hits), (c) a Teleost “all mRNA” database (80.1% hits) and (d) the *Danio rerio* Zv9 genome (6.3% hits). RNAseq reads from the nine individual samples were then aligned to this *de novo* assembled cDNA reference database. About 60% of the RNAseq reads could be mapped against the *de novo* assembled reference database. Statistical comparison between groups of fish SW1 vs. FMC and SW2 vs. FMC was done using HTseq and DESeq as described above under “strategy 1.” The set of differentially expressed contigs at *P* < 0.01 was cleaned by removing undefined mRNA sequences: 792 *Oncorhynchus mykiss* sequences; 40 *Plecoglossus altivelis* sequences; 10 *Ictalurus punctatus* sequences; 202 *Salmo salar* Sasaskin sequences; 11 *Gadus morhua* strain sequences; 5 *Fundulus grandis* transcripts; 2 *Anoplopoma fimbria* sequences; 2 *Scophthalmus maximus* sequences, and 391 blanks.

### Gene Ontology

From the cleaned set of differentially expressed contigs at *P* < 0.01 of strategy 2, contigs were selected that were associated with genes with apparent immune response function as determined by manually scanning gene ontology (GO) with Uniprot, QuickGO, Genecards, NCBI, Wikigene, and Wikipedia. These genes were identified in Uniprot and complete GO annotation on biological process level was analyzed in QuickGO. GO terms that reflected apparent immune response processes were collected per comparison SW1 vs. FMC and SW2 vs. FMC. GO differences were analyzed and significant differences identified applying Mann–Whitney *U* tests.

### Availability of Data and Material

The datasets (raw RNAseq reads) supporting the conclusions of this article are available in the NCBI GEO repository, accession numbers GSM2705950–GSM2705958^1^.

## Results

### Growth and Blood

**FIGURE 1:**
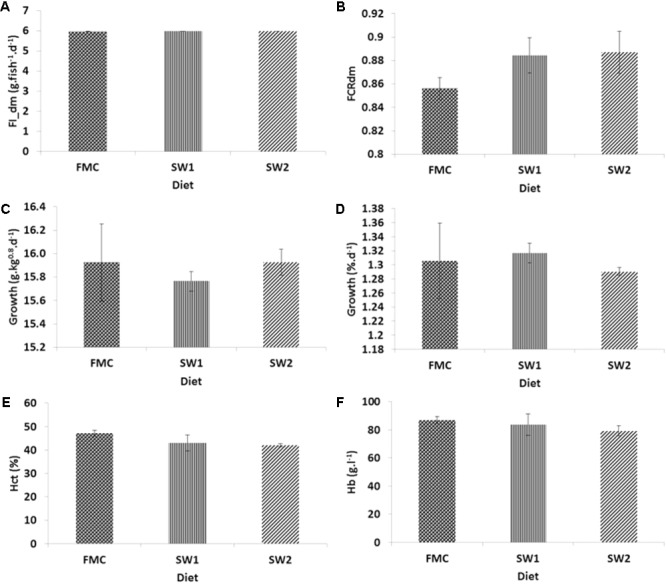
Atlantic salmon performance when fed with the FMC, SW1 and SW2 diets. **(A)** Feed intake in g.fish^-1^.d^-1^ on dry matter basis. **(B)** Feed conversion ratio (FCR) on dry matter basis. **(C)** Growth in metabolic body weight (g.kg^0.8^.d^-1^). **(D)** Specific growth rate (%.d^-1^). **(E)** Hct in %. **(F)** Hemoglobin (g.l^-1^). Fish fed with seaweed diets tended to have higher FCRdm values (*P* = 0.075) and less individual variation in growth.

Average feed intake was 5.97–5.98 g.dm.fish^-1^.d^-1^ and similar for all treatments (**Figure 1A**). Feed conversion on dry matter basis (FCRdm) between diets tended to differ but not significantly (*P* = 0.075; **Figure 1B**).

No differences in growth performance were detected between FMC, SW1, and SW2 fish (**Figures 1C,D**). Interestingly, fish fed with seaweed diets showed much less individual variation in growth performance than the fish fed the FMC control diet.

Fish fed FMC had average hematocrit (Hct) and hemoglobin (Hb) levels of 28.9 ± 9.9% and 62.9 ± 18.5 g.l^-1^, respectively, at the start of the experiment. Start values of both Hct and Hb were lower than values at the end of the experiment (*P* < 0.05), yet Hct and Hb did not differ between treatments at the end (*P* > 0.05) (**Figures 1E,F**).

### RNAseq Differential Expression After Mapping Reads Against NCBI *Salmo salar* Reference

The comparison SW1 vs. FMC yielded 28,312 expressed contigs associated with *S. salar* genes: 59% of the NCBI sequences. 144 of these contigs were differentially expressed: 74 up-regulated (51%) and 70 down-regulated (49%). Twenty contigs were considered as direct immune response genes of which nine genes (five up-regulated, four down-regulated) were specific for SW1 vs. FMC (**Table 4**).

**Table 4:** Differentially expressed immune response genes.

Specific for SW1	fc	
MHC class I (UBA) mRNA, UBA^∗^0901 allele	362	
T-cell receptor alpha chain V region 2B4 precursor putative mRNA	7.19	
MHC class I antigen (UBA) mRNA, UBA^∗^4001 allele	6.61	
Macrophage migration inhibitory factor putative mRNA	Inf	
MHC class I antigen (Sasa-UBA) mRNA, Sasa-UBA^∗^0902 allele	Inf	
BOLA class I histocompatibility antigen, alpha chain BL3-7 precursor putative mRNA	0.154	
MHC class I (UBA) mRNA, UBA^∗^0501 allele	0.131	
MHC class I mRNA	0.0467	
MHC class I (UBA) mRNA, UBA^∗^0701 allele	0.00130	

**Specific for SW2**	**fc**	

MHC class I antigen (Sasa-UBA) mRNA, Sasa-UBA^∗^3701 allele	288	
H-2 class II histocompatibility antigen gamma chain putative mRNA	137	
MHC class II antigen alpha chain (Sasa-DAA) mRNA	39.7	
MHC class II alpha mRNA	18.6	
Tumor necrosis factor receptor superfamily member 11B precursor putative mRNA	4.14	
CD209 antigen-like protein E putative mRNA	3.21	
Ig kappa chain V-IV region Len putative mRNA	2.86	
partial mRNA for MHC class II antigen beta chain (DAB gene)	Inf	
MHC class I antigen (Sasa-UBA) mRNA, Sasa-UBA^∗^1402 allele	Inf	
Class I histocompatibility antigen, F10 alpha chain precursor putative mRNA	0.254	
MHC class I (UBA) mRNA, UBA^∗^0501 allele	0.131	
MHC class I antigen (Sasa-UBA) mRNA, Sasa-UBA^∗^3501 allele	0.125	
Proliferating cell nuclear antigen putative mRNA	0.113	
MHC class I (UBA) mRNA, UBA^∗^1501 allele	0.00567	
Anamorsin putative mRNA	0	

**Common SW1-SW2**	**SW1 (fc)**	**SW2 (fc)**

MHC class II antigen beta chain (DAB gene)	309	171
MHC-Sasa class II (clone c144)	54.7	7.76
MHC class I (UBA) mRNA, UBA^∗^0201 allele	36.8	35.0
MHC class I antigen (Sasa-UBA) mRNA	Inf	Inf
MHC class I antigen (Sasa-UBA) mRNA, Sasa-UBA^∗^0202 allele	Inf	Inf
MHC class II antigen alpha chain (Sasa-DAA) mRNA	Inf	Inf
HLA class II histocompatibility antigen, DQW1.1 beta chain precursor putative mRNA	Inf	Inf
CD209 antigen-like protein E putative mRNA	Inf	Inf
MHC class I (UBA) mRNA, UBA^∗^1401 allele	0.350	0.351
MHC-Sasa class II B (clone c22)	0.276	0.003
MHC class I antigen (Sasa-UBA) mRNA, Sasa-UBA^∗^3901 allele	0.00349	0

*Differentially expressed immune response genes at *P* < 0.05, specific for the comparison SW1 vs. FMC, SW2 vs. FMC and those in common as a result of strategy 1; Mapping reads against NCBI *Salmo salar* reference. Expression is given by fold change (fc) and Inf indicates that expression was unique for the particular seaweed.*

The comparison SW2 vs. FMC yielded 28,189 expressed contigs associated with *S. salar* genes: 58% of the NCBI sequences. Two hundred and forty-six of these contigs were differentially expressed: 136 up-regulated (55%) and 110 down-regulated (45%). Twenty-six contigs were considered as direct immune response genes of which 15 genes (nine up-regulated, six down-regulated) were specific for SW2 vs. FMC (**Table 4**).

Aligning vs. NCBI *S. salar* sequences and comparing SW1 vs. FMC and SW2 vs. FMC revealed 54 common differentially expressed contigs. Expression of all these contigs was in the same direction, either up- or down-regulated. Eleven contigs (eight up-regulated, three down-regulated) were considered as direct immune response genes representing the common genes in the immunomodulatory effects of the tested dietary seaweeds (**Table 4**).

### RNAseq Differential Expression and Gene Ontology After Mapping Reads Against *de Novo* Assembled *Salmo salar* Reference

The comparison SW1 vs. FMC yielded 1,951 differentially expressed contigs: 2.23% of the total of 87,600 contigs. Expression of 1,092 contigs (56%) was up-regulated, of 859 contigs (44%) down-regulated. On basis of more the stringent criterion of *P* < 0.01, 736 contigs were selected for further analyses.

The comparison SW2 vs. FMC yielded 4,350 differentially expressed contigs: 4.97% of the total of 87,600 contigs. Expression of 1,485 contigs (34%) was up-regulated, of 2,865 contigs (66%) down-regulated. On basis of the more stringent criterion of *P* < 0.01, 1,703 contigs were selected for further analyses.

BLAST searching against four reference databases and comparing SW1 vs. FMC and SW2 vs. FMC revealed 224 common differentially expressed contigs. Expression of these contigs was in the same direction, except for five: Glutamate dehydrogenase 1, Serine incorporator 1 and two *Oncorhynchus mykiss* contig sequences, of which expression was in opposite direction, as well as for *NF-kappa-B inhibitor alpha (S. salar)*.

Interestingly, contig 86142 had by far the highest fold change (fc) for both SW1 vs. FMC and SW2 vs. FMC comparisons with fc 2,550 and 2,968, respectively. This contig was mapped against the *Oncorhynchus mykiss* mRNA sequence with gene identifier 299677870 that resulted from a characterization of the rainbow trout transcriptome using Sanger and 454-pyrosequencing approaches by Salem et al. (2010). With a BLASTn search, a 98% homology was found aligning this sequence with *PREDICTED: Salmo salar integrin alpha-2-like* (LOC106561807).

For the comparison SW1 vs. FMC, 67 contigs were related to immune response genes. Specific for this comparison were 43 immune response genes: 32 up-regulated, 11 down-regulated (Supplementary material). Twenty-four genes were shared with the comparison SW2 vs. FMC: 12 up-regulated, 12 down-regulated (Supplementary material). In total 39 genes contributed to the pool of 168 GO terms for this comparison (84 specific and 84 in common with comparison SW2 vs. FMC).

For the comparison SW2 vs. FMC, 156 contigs were related to immune response genes. Specific for this comparison were 132 genes: 67 up-regulated, 65 down-regulated (Supplementary material). Of course, 24 genes were shared with the comparison SW1 vs. FMC, but now with 11 up-regulated and 13 down-regulated because *NF-kappa-B inhibitor alpha (S. salar)* was down-regulated and not up-regulated like for SW1 vs. FMC (Supplementary material). In total 82 genes contributed to the pool of 256 GO terms for this comparison (172 specific).

**FIGURE 2:**
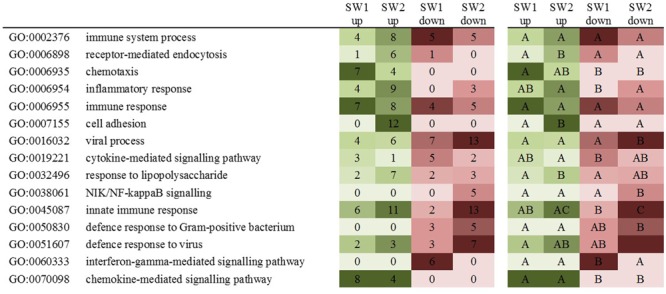
Heat map of the 15 main biological processes indicating the differences between diet comparisons. Shown are GO terms and descriptions; seaweed (SW) diets 1 and 2 scores of up- and down-regulated genes. Also indicated the significant differences (*P* < 0.05).

The number of GO terms was narrowed down removing those with less than five scores, which left us with the 15 most important GO terms (**Figure 2**). Six of these GO terms did not show differences between comparisons. More regular GO terms such as *immune system process* (GO:0002376), *immune response* (GO:0006955), and *viral process* (GO:0016032) had as many scores for SW1 vs. FMC as for SW2 vs. FMC for both up- and down-regulated genes. *Chemotaxis* (GO:0006935) and *chemokine-mediated signaling pathway* (GO:0070098) only contained up-regulated gene expression for both comparisons, expression of genes involved in the *defense response to Gram-positive bacterium* (GO:0050830) was down-regulated for both comparisons. Other GO terms were more abundant or even specific for either the SW1 vs. FMC or SW2 vs. FMC comparison. Abundant or specific for the SW1 vs. FMC comparison were *cytokine-mediated signaling pathway* (GO:0019221) and the *interferon-gamma-mediated signaling pathway* (GO:0060333). More terms were abundant or specific for the SW2 vs. FMC comparison: *receptor-mediated endocytosis* (GO:0006898), *inflammatory response* (GO:0006954), *cell adhesion* (GO:0007155), and *response to lipopolysaccharide* (GO:0032496) all up-regulated*; innate immune response* (GO:0045087) up- and down-regulated; *NIK/NF-kappaB signaling* (GO:0038061) and *defense response to virus* (GO:0051607) down-regulated.

## Discussion

Seaweeds may represent immuno-stimulants that could be used as health-promoting fish feed components thereby offering an alternative for the use of antibiotics. This study was performed to gain insights into the immunomodulatory effects of dietary seaweeds in Atlantic salmon. Specifically tested were 10% inclusion levels of *Laminaria digitata* (SW1) and a commercial blend of seaweeds (Oceanfeed^®^) (SW2) against a fishmeal based control diet (FMC). By RNAseq of the head kidney transcriptome of LPS challenged fish representing all three experimental groups, we could determine the common immunomodulatory effects of dietary seaweeds, but also the specific immunomodulatory effects of *Laminaria digitata* and of the commercial blend of seaweeds.

### No Major Difference in Performance

From our results it can be concluded that the inclusion of dietary seaweeds did not lead to major differences in performance of Atlantic salmon: growth and blood Hct and Hb were similar between experimental groups. The FCR of the fish fed with dietary seaweeds was not significantly different. Growth variation in the fish fed with dietary seaweeds, however, was lower which would lead to a more uniform market size. Similarly as in previously performed studies, dietary seaweeds did not affect growth performance of rainbow trout nor seabass at inclusion levels of 10% (*Oncorhynchus mykiss*: Soler-Vila et al., 2009, *Dicentrarchus labrax*; Valente et al., 2006; Peixoto et al., 2016). Several other studies did report on growth improvement by dietary seaweed supplementation, but the variety of both fish and seaweed species, and their inclusion levels, is extensive which makes it complex to generalize conclusions. Anyway, the absence of major difference in the performance of Atlantic salmon would pave the way for dietary seaweed application if clear health-promoting effects would exist.

### Nutrigenomic Approach Investigating Dietary Immunomodulation

Recent studies have provided more insights into the dietary immunomodulation of gene expressions since the review by Tacchi et al. (2011). Several studies have a similar nutrigenomic approach as our study and support immune enhancement on basis of up-regulated gene expressions in Atlantic salmon. Martinez-Rubio et al. (2012, 2014) reported on the dietary immunomodulation of gene expressions in the heart in response to immune challenging. Núñez-Acuña et al. (2015) investigated the effects of plant-derived additives on the skin and heart kidney transcriptome in sea lice-infested Atlantic salmon. Eslamloo et al. (2017) investigated whether vegetable and fish oils can alter antiviral responses of salmon macrophage-like cells by transcriptomic profiling but found similar activation of the immune-related pathways and functions between experimental groups. Caballero-Solares et al. (2017) concluded that plant-based diets may enhance the immune response on basis of increased expression of transcripts involved in the synthesis of pro-inflammatory eicosanoids and chemotaxis. Our study supports the general immune enhancement of dietary seaweeds but also shows that the seaweed specific effects are important and may vary significantly.

### Common Immunomodulatory Effects of Dietary Seaweeds

From the *de novo* assembled reference we learned that GO terms that represented common immunomodulatory effects were referring to quite general processes (*immune system process, immune response*, and *viral process*) but also more specifically to c*hemotaxis, chemokine-mediated signaling pathway* and *defense response to Gram-positive bacterium*. From the direction of regulation of the genes that were representing these GO terms, we can conclude that the common immunomodulatory effects of the dietary seaweeds in our study improved *chemotaxis* and the *chemokine-mediated signaling pathway*, and reduced the *defense response to Gram-positive bacterium*. Thus, based on the regulation of these immune genes, fish fed with seaweeds have a more efficient immune response. The infection site is more efficiently identified and antigens are more efficiently processed and presented.

The contig 86142 with fc values of 2,550 and 2,968 for SW1 vs. FMC and SW2 vs. FMC comparisons, respectively, that had a 98% homology with the *predicted Salmo salar integrin alpha-2-like*, may certainly represent a key marker gene of the LPS immune response in salmonids. This gene is predicted to express a subunit of the heterodimeric integral membrane glycoprotein integrin which is involved in cell adhesion and cell-surface mediated signaling of T cells (the NKT cells), NK cells, fibroblasts and platelets. *Integrin alpha-2* is known to show up-regulated expression at the attachment site of salmon louse in order to regulate a cell proliferation response (Robledo et al., unpublished) but our results show that its up-regulated expression is not restricted to response to a skin wound.

Read annotation to *S. salar* NCBI sequences revealed predominantly MHC class I and II and other genes involved in antigen processing and presentation. Common for both SW1 vs. FMC and SW2 vs. FMC was also the expression of *CD209 antigen-like protein E* which is a putative pathogen-recognition receptor that may mediate the endocytosis of pathogens (Dettleff et al., 2017; Rozas-Serri et al., 2018). The particular transcript that was common for both groups was expressed only in the SW1 and SW2 groups (two and one fish, respectively) and not in the FMC controls. *CD209 antigen-like protein* enhances the expression of Toll-like receptors (TLR; Geijtenbeek and Gringhuis, 2009) which in our study was only apparent for *TLR8* for diet SW2.

Mapping reads against the *de novo* assembled *S. salar* reference revealed more important genes that were differentially expressed for SW1 vs. FMC and SW2 vs. FMC. Highly up-regulated was the *Ig heavy chain V-III region HPC76 Fragment (M. musculus)*, which emphasizes the up-regulation of antigen recognition in fish fed with dietary seaweeds. Also up-regulated is *T-bet (O. mykiss)* which in Atlantic salmon plays an important role in Th1 T-helper cell differentiation (Kumari et al., 2015). Furthermore, among the up-regulated genes is *C-C motif chemokine 19 (M. musculus)* that may direct the improvement in chemotaxis and the chemokine-mediated signaling pathway which were identified through GO as important common pathways. The up-regulated *nuclear receptor subfamily 1 group D member 2 (M. musculus)* is a transcriptional repressor that regulates genes involved in the inflammatory response (Lam et al., 2013). The up-regulated *Secretory phospholipase A2 receptor (P. abelii)* is involved in cytokine production and *Src-like-adapter 2 (H. sapiens)* negatively regulates T-cell receptor signaling (Holland et al., 2001).

*NF-kappa-B inhibitor alpha (S. salar)* is both up- and down-regulated and inhibits NF-kappa-B as mediator impacting processes such as apoptosis, proliferation, differentiation, and development (Lee et al., 2014). Then, in SW1 fish NF-kappa-B activity is inhibited while in SW2 fish NF-kappa-B activity is stimulated. Besides *NF-kappa-B inhibitor alpha (S. salar)*, also *glutamate dehydrogenase 1* and *serine incorporator 1* expression was in opposite direction for both experimental seaweed diets.

Among the down-regulated genes is *N-acetylmuramoyl-L-alanine amidase (M. musculus)* which is involved in the reduction of the *defense response against Gram-positive bacterium* (e.g., Zhang et al., 2016 for turbot). Also down-regulated are three transcripts associated with *Heat shock cognate 71 kDa protein* (*G. gallus, O. latipes*, and *S. oedipus*), which binds LPS and then mediates the LPS-induced inflammatory response including TNF secretion by monocytes, and the cytokine receptor subunit *Cytokine receptor-like factor 1 (H. sapiens)*.

### Specific Immunomodulatory Effects of 10% *Laminaria digitata* Inclusion

Annotation of reads to the *de novo* assembled reference showed that specific immunomodulatory effects of dietary *Laminaria digitata* include mostly down-regulated gene expression in the *cytokine-mediated signaling pathway*, specifically the *interferon-gamma-mediated signaling pathway*. The interferon-gamma-receptors are internalized after binding and through clathrin-coated pits delivered to the sorting endosome (Blouin and Lamaze, 2013). The specific modulatory effects of *Laminaria digitata* included the reduction of this process.

Besides MHC class I genes and a T-cell receptor, specific for the SW1 immunomodulatory effect was the expression of *macrophage migration inhibitory factor* (MIF), which is a widely expressed pro-inflammatory cytokine inhibiting the random migration of macrophages (Jin et al., 2007) which again emphasizes the importance of the *cytokine-mediated signaling pathway*.

Mapping reads against the *de novo* assembled *S. salar* reference also revealed a MHC I gene as very important and specific for SW1 vs. FMC: *Major histocompatibility complex class I-related gene protein (M. musculus)* which was up-regulated at fc 1,681. Second in line was *sorting nexin-18 (S. salar)*, involved in endocytosis (Park et al., 2010), with up-regulated expression at fc 71.3. Other important up-regulated genes were *C-X-C motif chemokine 13 (H. sapiens)*, chemotactic for B-lymphocytes, and *dedicator of cytokinesis protein 2 (O. mykiss)* also involved in chemotaxis by making arrangements for lymphocyte migration in response of chemokines.

Down-regulated genes include *class I histocompatibility antigen, F10 alpha chain (G. gallus)* and *class I histocompatibility antigen, A9/A9 alpha chain (C. familiaris)*, involved in the presentation of foreign antigens to the immune system. Also down-regulated is the *signal transducer and activator of transcription 1* or *STAT1* (two transcripts: *M. musculus* and alpha/beta; *H. sapiens*), which stimulates the expression of genes in response to an interferon signal (Skjesol et al., 2010). STAT1 and the *gamma-interferon-inducible lysosomal thiol reductase precursor (S. salar)* both contribute to the down-regulated *interferon-gamma-mediated signaling pathway*.

### Specific Immunomodulatory Effects of 10% Inclusion of a Commercial Blend of Seaweeds

Annotation of reads to the *de novo* assembled reference showed that specific immunomodulatory effects of including a commercial blend of seaweeds, in this case Oceanfeed^®^, include up-regulated gene expression in *receptor-mediated endocytosis* and *cell adhesion*, and *response to lipopolysaccharide* and *inflammatory response*: processes that are supposedly improved by the commercial blend of seaweeds. Biological processes such as *NIK/NF-kappa-B signaling* and *defense response to virus* were reduced in their functioning. Genes involved in the *innate immune response* were both up- and down-regulated.

By far most genes that were differentially regulated were associated with the specific modulatory effects of Oceanfeed^®^. Similar to *Laminaria digitata*, read annotation to the *S. salar* NCBI sequences revealed *MHC class I* genes being up-and downregulated, *MHC class II* genes were up-regulated. Also up-regulated was *tumor necrosis factor receptor superfamily member 11B precursor* that binds cytokine tumor necrosis factor causing cell death, which is in line with the down-regulation of *anamorsin* that is involved in the negative control of cell death upon cytokine withdrawal. Other up-regulated genes were *Ig kappa chain V-IV region Len* which is involved in antigen binding and *CD209 antigen-like protein E* involved in endocytosis. *Proliferating cell nuclear antigen* is essential for DNA replication and its expression was down-regulated.

Mapping reads against the *de novo* assembled *S. salar* reference revealed strongly up-regulated expression of important immune receptors such as *T-cell receptor gamma (S. salar)*; *cholecystokinin receptor type A (M. musculus* and *C. familiaris)*; *Interleukin-13 receptor alpha-2 (S. salar)*; *scavenger receptor cysteine-rich type 1 protein M130 (M. musculus)* and *protein M160 (H. sapiens)*; *transferrin receptor protein 1 (C. familiaris)*; *tumor necrosis factor receptor superfamily member 11B (H. sapiens)*; *macrophage mannose receptor 1 (M. musculus)*; *chemokine receptor-like 1 (O. mykiss)*; *C-X-C chemokine receptor type 1 (O. cuniculus)* and the *nuclear receptor subfamily 5 group A member 2 (H. sapiens)*, which agrees with the increased responsiveness as determined by gene ontology. Other important up-regulated genes include *tumor necrosis factor-inducible gene 6 protein (O. cuniculus)* and the *toll-like receptor 8 (S. salar)* that, like other members of the TLR family, plays a fundamental role in pathogen recognition and activation of innate immunity by production of cytokines (Rebl et al., 2010; Pietretti and Wiegertjes, 2014). Important down-regulated genes include cytokine-related genes, such as the *tumor necrosis factor alpha-induced protein 3 (O. mykiss)* and the *lipopolysaccharide-induced tumor necrosis factor-alpha (S. salar)*; the *cytokine inducible SH2-containing protein (S. salar)* and the *FL cytokine receptor precursor (S. salar)*, and the *proteasome activator complex subunit 4-like (O. niloticus)*. Down-regulation of the *myxovirus resistance 1 (S. salar)* illustrates the reduced viral immune response.

### RNAseq Validation and Analyses on the Physiological Process Level

RNAseq data in this study were not validated by RT-qPCR. Unlike with microarray data, the verification of RNAseq data by RT-qPCR is often done but not necessarily required. RNAseq and RT-qPCR are two different methods with a different dynamic range of sensitivities. RNAseq has a much broader linear range than microarrays and the statistical analyses by DESeq are very stringent. RT-qPCR itself is often not even well validated according to MIQE guidelines. Validation using RT-qPCR on the same RNA samples as assayed in the RNA-seq analysis only validates the technology, it does not validate the conclusion about the treatments or conditions (Fang and Cui, 2011). Moreover, RNAseq represents a high-throughput transcriptomic approach, specifically useful when analysing on the level of physiological processes (e.g., Palstra et al., 2015; Rurangwa et al., 2015) which was also the main aim of this study. This paper is aimed to be a first step toward a project on the immune modulating effects of seaweeds in which this study can be followed up by dedicated RT-qPCR studies to validate the results obtained on a new and larger set of samples.

## Conclusion

In conclusion, Atlantic salmon fed with dietary seaweeds showed more homogenous growth but was not different in FCR. In general no major differences in performance were found in comparison with fishmeal fed fish. However, the dietary seaweeds provoke a more efficient immune response, which involves more efficient identification of the infection site and processing and presentation of antigens. Specific *Laminaria digitata* effects included reduction of *interferon-gamma-mediated signaling*. The commercial blend of seaweeds improved immune processes such as *receptor-mediated endocytosis* and *cell adhesion*, and increased the expression of genes involved in *response to lipopolysaccharide* and *inflammatory response*. It can be concluded that the inclusion of seaweeds such as *Laminaria digitata*, can have important modulatory effects on the immune capacity of Atlantic salmon resulting in a more efficient immune response.

## Data Availability

The raw data supporting the conclusions of this manuscript will be made available by the authors, without undue reservation, to any qualified researcher.

## Author Contributions

AP and JK: conception and design of the study. AP, JK, ABG, and RD: acquisition of data. AP, JK, and RD: analysis and interpretation of data. AP, JK, RD, and MP: drafted the manuscript and reviewed. All authors read and approved the final manuscript.

## Conflict of Interest Statement

RD was employed by company ZF-screens B.V. The other authors declare that the research was conducted in the absence of any commercial or financial relationships that could be construed as a potential conflict of interest.

## Footnotes

1 https://www.ncbi.nlm.nih.gov/geo/
