# Supplementary material for: Immunomodulatory Effects of Dietary Seaweeds in LPS Challenged Atlantic Salmon Salmo salar as Determined by Deep RNA Sequencing of the Head Kidney Transcriptome
Source: Front Physiol. 2018 Jun 1;9:625. doi: 10.3389/fphys.2018.00625 (PMC5992350; doi:10.3389/fphys.2018.00625)
Supplement: Supplementary file 1 [file Table_1.docx]

**Supplementary material:** Differentially expressed immune response genes (P<0.01), specific for the comparison SW1 *vs*. FMC, SW2 *vs*. FMC and those in common as a result of strategy 2; Mapping reads against de novo assembled *Salmo salar* reference. Expression is given by fold change (fc) and Inf indicates that expression was unique for the particular comparison. Gene descriptions in bold indicate genes that contribute to immune GO analysis.

| **specific for SW1 (N=43: 32 up, 11 down)** | **fc** |  |
| --- | --- | --- |
| Protein YLR162W (*S. cerevisiae* strain ATCC 204508 / S288c) | Inf |  |
| **Interferon-induced 35 kDa protein homolog (*S. salar*)** | Inf |  |
| **Major histocompatibility complex class I-related gene protein (*M. musculus*)** | 1681 |  |
| Sorting nexin-18 (*S. salar*) | 71.3 |  |
| GTPase IMAP family member 8 (*H. sapiens*) | 6.39 |  |
| Sialic acid-binding Ig-like lectin 12 precursor (*S. salar*) | 5.99 |  |
| **C-X-C motif chemokine 13 (*H. sapiens*)** | 5.74 |  |
| SH3 domain-binding protein 5 (*H. sapiens*) | 4.92 |  |
| Granulins (*R. norvegicus*) | 3.57 |  |
| **Dedicator of cytokinesis protein 2 (*O. mykiss*)** | 3.13 |  |
| MAP kinase-interacting serine/threonine-protein kinase 2 (*X. laevis*) | 3.04 |  |
| Catenin beta-1 (*S. salar*) | 2.75 |  |
| **Complement component C8 gamma chain (*H. sapiens*)** | 2.64 |  |
| **Perforin-1 (*H. sapiens*)** | 2.63 |  |
| Fructose-bisphosphate aldolase B (*D. rerio*) | 2.60 |  |
| **Sec1 family domain-containing protein 1 (*S. salar*)** | 2.56 |  |
| **Cyclin-dependent kinase inhibitor 1B (*H. sapiens*)** | 2.56 |  |
| TSC22 domain family protein 3 (*P. abelii*) | 2.51 |  |
| **polyprotein, Baboon endogenous virus strain M7** | 2.49 |  |
| **C-C chemokine receptor type 9 (*M. musculus*)** | 2.38 |  |
| **Adenosine receptor A2a (*E. caballus*)** | 2.33 |  |
| **Annexin A11 (*S. salar*)** | 2.31 |  |
| **vascular cell adhesion molecule-like protein (*O. mykiss*)** | 2.28 |  |
| **Leukemia inhibitory factor receptor (*C. familiaris*)** | 2.24 |  |
| **Vascular cell adhesion protein 1 (*C. familiaris*)** | 2.22 |  |
| Uromodulin (*B. taurus*) | 2.20 |  |
| **Nuclear receptor subfamily 1 group D member 1 (*H. sapiens*)** | 2.20 |  |
| Perforin-1 (*R. norvegicus*) | 2.19 |  |
| **C-C chemokine receptor type 9 (*O. aries*)** | 2.16 |  |
| **Lysosome-associated membrane glycoprotein 3 (*H. sapiens*)** | 2.13/2.33 |  |
| **Basic leucine zipper transcriptional factor ATF-like 3 (*H. sapiens*)** | 2.09 |  |
| Transcription factor Maf (*D. rerio*) | 2.01 |  |
| **Ig heavy chain V region 3 (*C. auratus*)** | 0.486 |  |
| **SAM domain and HD domain-containing protein 1 (*D. rerio*)** | 0.472 |  |
| **Ankyrin repeat and FYVE domain-containing protein 1 (*M. musculus*)** | 0.415 |  |
| Calreticulin (*B. taurus*) | 0.384 |  |
| **Signal transducer and activator of transcription 1-alpha/beta (*H. sapiens*)** | 0.265 |  |
| Inositol 1,4,5-trisphosphate receptor type 1 (*B. taurus*) | 0.223 |  |
| **class I histocompatibility antigen, A9/A9 alpha chain (*C. familiaris*)** | 0.193 |  |
| **Gamma-interferon-inducible lysosomal thiol reductase precursor (*S. salar*)** | 0.188 |  |
| **Signal transducer and activator of transcription 1 (*M. musculus*)** | 0.142 |  |
| **Class I histocompatibility antigen, F10 alpha chain (*G. gallus*)** | 0.139 |  |
| Mitochondrial ubiquitin ligase activator of NF-kB (*S. salar*) | 0.000 |  |
|  |  |  |
| **specific for SW2 (N=132: 67 up, 65 down)** | **fc** |  |
| **MHC class I heavy chain precursor (*O. mykiss*)** | Inf |  |
| T-cell receptor gamma (*S. salar*) | 13.0 |  |
| Acyl-coenzyme A thioesterase 8 (*S. salar*) | 11.5 |  |
| Histone H2B (*S. trutta*) | 6.88 |  |
| Coatomer subunit beta (*B. taurus*) | 6.10 |  |
| **Apolipoprotein D (*C. porcellus*)** | 6.09 |  |
| Tyrosine-protein kinase transforming protein erbB, Avian leukosis virus | 5.59 |  |
| **Prospero homeobox protein 1 (*M. musculus*)** | 5.43 |  |
| **Tumor necrosis factor-inducible gene 6 protein (*O. cuniculus*)** | 5.42 |  |
| netrin receptor UNC5D-like, transcript variant X1 (*D. rerio*) | 5.08 |  |
| **Cholecystokinin receptor type A (*M. musculus*)** | 5.05 |  |
| **Phosphatidylinositol 3,4,5-trisphosphate 5-phosphatase 2A (*D. rerio*)** | 4.91 |  |
| Glutamyl aminopeptidase (*M. musculus*) | 4.81 |  |
| **Cytoplasmic dynein 1 heavy chain 1 (*H. sapiens*)** | 4.36 |  |
| Interleukin-13 receptor alpha-2 (*S. salar*) | 4.28 |  |
| Bone morphogenetic protein 1 precursor (*S. salar*) | 4.16 |  |
| **cyclin-dependent kinase 19-like (*D. rerio*)** | 3.97 |  |
| **Scavenger receptor cysteine-rich type 1 protein M130 (*M. musculus*)** | 3.77 |  |
| Granzyme-like protein 1 (*R. norvegicus*) | 3.62 |  |
| Pleckstrin homology domain-containing family A member 6 (*M. musculus*) | 3.42 |  |
| **Scavenger receptor cysteine-rich type 1 protein M160 (*H. sapiens*)** | 3.36 |  |
| Spectrin beta chain, non-erythrocytic 2 (*R. norvegicus*) | 3.26 |  |
| GTPase IMAP family member 4 (*H. sapiens*) | 3.15 |  |
| **TLR8-like protein (*S. salar*)** | 3.08 |  |
| **Lysosomal-trafficking regulator (*B. taurus*)** | 2.94 |  |
| Fatty acid-binding protein, adipocyte (*S. salar*) | 2.88 |  |
| **Cholecystokinin receptor type A (*C. familiaris*)** | 2.80 |  |
| **Transferrin receptor protein 1 (*C. familiaris*)** | 2.66 |  |
| **Immune-responsive gene 1 protein homolog (*H. sapiens*)** | 2.66 |  |
| Matrix metalloproteinase-9 (*M. musculus*) | 2.66 |  |
| **Matrix metalloproteinase-14 (*O. cuniculus*)** | 2.63 |  |
| Integrin alpha-Iib (*M. musculus*) | 2.56/2.95 |  |
| **Tumor necrosis factor receptor superfamily member 11B (*H. sapiens*)** | 2.48 |  |
| **5'-AMP-activated protein kinase subunit gamma-2 (*H. sapiens*)** | 2.47 |  |
| **AP-3 complex subunit delta-1 (*B. taurus*)** | 2.43 |  |
| Fasciculation and elongation protein zeta-2 (*M. musculus*) | 2.33 |  |
| **Matrix metalloproteinase-9 (*H. sapiens*)** | 2.27 |  |
| **Integrin beta-3 (*M. musculus*)** | 2.27 |  |
| **Macrophage mannose receptor 1 (*M. musculus*)** | 2.24 |  |
| Protein-tyrosine sulfotransferase 1 (*D. rerio*) | 2.22 |  |
| Chemokine receptor-like 1 (*O. mykiss*) | 2.20 |  |
| **Vascular cell adhesion protein 1 (*R. norvegicus*)** | 2.17 |  |
| CD226 antigen (*M. mulatta*) | 2.15 |  |
| Transcription factor MafB (*S. salar*) | 2.14 |  |
| Spectrin beta chain, non-erythrocytic 1 (*M. musculus*) | 2.14/2.58 |  |
| **Complement component C7 (*S. scrofa*)** | 2.12 |  |
| Tumor necrosis factor alpha-induced protein 2 (*H. sapiens*) | 2.11 |  |
| Glutamyl aminopeptidase (*H. sapiens*) | 2.05 |  |
| Lysosome membrane protein 2 (*R. norvegicus*) | 2.04 |  |
| **Fibronectin (*X. laevis*)** | 2.04/2.86 |  |
| **Matrix metalloproteinase 14 (*R. norvegicus*)** | 2.02 |  |
| Gamma-glutamyltransferase 5 (*R. norvegicus*) | 2.02 |  |
| Putative acyl-CoA dehydrogenase AidB (*E. coli* strain K12) | 2.01 |  |
| **Matrix metalloproteinase-14 (*B. taurus*)** | 1.99 |  |
| Amyloid beta A4 protein (*T. rubripes*) | 1.98 |  |
| **Charged multivesicular body protein 5 (*S. salar*)** | 1.97 |  |
| Lysosome membrane protein 2 (*M. musculus*) | 1.96 |  |
| **C-X-C chemokine receptor type 1 (*O. cuniculus*)** | 1.96 |  |
| **CD83 antigen precursor (*S. salar*)** | 1.93 |  |
| **P-selectin (*H. sapiens*)** | 1.92 |  |
| **C-C motif chemokine 4 (*M. mulatta*)** | 1.92 |  |
| **Nuclear receptor subfamily 5 group A member 2 (*H. sapiens*)** | 1.92/2.90/5.05 |  |
| **C-type lectin domain family 4 member E (*H. sapiens*)** | 1.90/2.03 |  |
| **ATP-binding cassette sub-family A member 1 (*M. musculus*)** | 1.88 |  |
| **Complement component C7 (*H. sapiens*)** | 1.86 |  |
| Metalloreductase STEAP4 (*H. sapiens*) | 1.86/1.88 |  |
| Nucleolysin TIAR (*S. salar*) | 1.72 |  |
| **C-type lectin domain family 4 member M (*G. gorilla gorilla*)** | 0.535 |  |
| **FYN-binding protein (*S. salar*)** | 0.493 |  |
| Arrestin domain-containing protein 3 (*H. sapiens*) | 0.49 |  |
| Collagen alpha-1 I chain (*C. pyrrhogaster*) | 0.489 |  |
| **Megakaryocyte-associated tyrosine-protein kinase (*H. sapiens*)** | 0.487 |  |
| Ankyrin repeat and SOCS box protein 8 (*B. taurus*) | 0.478 |  |
| **Lysosome-associated membrane glycoprotein 1 (*B. taurus*)** | 0.467 |  |
| **Complement decay-accelerating factor Fragment (*P. pygmaeus*)** | 0.464 |  |
| Myotubularin-related protein 3 (*H. sapiens*) | 0.461 |  |
| Mixed lineage kinase domain-like protein (*S. salar*) | 0.458 |  |
| **Non-histone chromosomal protein H6 (*S. salar*)** | 0.457 |  |
| DNA replication licensing factor mcm2 (*S. salar*) | 0.456 |  |
| Ubiquitin-conjugating enzyme E2 A (*S. salar*) | 0.456 |  |
| **Hypoxia-inducible factor 1-alpha (*G. gallus*)** | 0.449 |  |
| **BCL2/adenovirus E1B 19 kDa protein-interacting protein 3-like (*B. taurus*)** | 0.421/0.364 |  |
| Double-stranded RNA-binding protein Staufen homolog 1 (*S. salar*) | 0.417 |  |
| **Actin-related protein 2/3 complex subunit 5 (*S. salar*)** | 0.415 |  |
| enhancer of polycomb homolog 1-like, transcript variant X3 (*D. rerio*) | 0.414 |  |
| T-cell acute lymphocytic leukemia protein 1 homolog (*D. rerio*) | 0.412/0.404/0.314 |  |
| immunoglobulin heavy chain variable region (*S. alpinus*) | 0.408 |  |
| Cyclin-dependent kinase 4 inhibitor B (*H. sapiens*) | 0.401 |  |
| **Adenylate cyclase type 7 (*S. salar*)** | 0.399 |  |
| Interleukin-31 receptor A (*S. salar*) | 0.398 |  |
| **GATA-binding factor 3 (*X. laevis*)** | 0.397 |  |
| **E3 ubiquitin-protein ligase TRIM63 (*H. sapiens*)** | 0.39 |  |
| **TRAF-type zinc finger domain-containing protein 1 (*S. salar*)** | 0.384 |  |
| **baculoviral IAP repeat-containing protein 6-like (*O. latipes*)** | 0.380 |  |
| Voltage-dependent anion-selective channel protein 1 (*S. salar*) | 0.379/0.346 |  |
| **E3 ISG15--protein ligase HERC5 (*H. sapiens*)** | 0.373 |  |
| **Ig mu chain C region membrane-bound form (*I. punctatus*)** | 0.370 |  |
| Nuclear cap binding protein subunit 2 (*Salmo salar*) | 0.363 |  |
| **3-phosphoinositide-dependent protein kinase 1-like, transcript variant X2 (*D. rerio*)** | 0.359 |  |
| Proteasome subunit beta type-2 (*S. salar*) | 0.351 |  |
| NudC domain-containing protein 1 (*S. salar*) | 0.350 |  |
| **Heterogeneous nuclear ribonucleoprotein U-like protein 1 (*S. salar*)** | 0.346 |  |
| **Krueppel-like factor 2 (*M. musculus*)** | 0.335 |  |
| **Proteasome subunit alpha type-3 (*S. salar*)** | 0.335 |  |
| T-box transcription factor TBX6L (*D. rerio*) | 0.319 |  |
| **Engulfment and cell motility protein 1 (*S. salar*)** | 0.318 |  |
| Mucosa-associated lymphoid tissue lymphoma translocation protein 1 (*S. salar*) | 0.313 |  |
| tumor protein p53-inducible nuclear protein 2-like (*D. rerio*) | 0.309 |  |
| **Neurogenic locus notch homolog protein 1 (*H. sapiens*)** | **0.307** |  |
| Dual specificity protein phosphatase 7 (*S. salar*) | 0.306 |  |
| Fish-egg lectin (*C. carpio*) | 0.295 |  |
| **Myeloperoxidase (*M. musculus*)** | 0.291 |  |
| Lipopolysaccharide-induced tumor necrosis factor-alpha (*S. salar*) | 0.291 |  |
| **Serine/threonine-protein kinase 10 (*D. rerio*)** | 0.290 |  |
| **Transcription factor RelB (*S. salar*)** | 0.285 |  |
| Glyceraldehyde-3-phosphate dehydrogenase (*P. abelii*) | 0.282 |  |
| Glyceraldehyde-3-phosphate dehydrogenase (*D. rerio*) | 0.278 |  |
| ATP-binding cassette sub-family G member 4 (*H. sapiens*) | 0.256 |  |
| **cytokine inducible SH2-containing protein (*S. salar*)** | 0.256/0.215 |  |
| **cullin-5-like (*T. rubripes*)** | 0.246 |  |
| Calcium/calmodulin-dependent protein kinase type II gamma chain (*S. salar*) | 0.244 |  |
| Butyrophilin subfamily 1 member A1 (*S. salar*) | 0.242 |  |
| **interleukin-1 beta (*S. salar*)** | 0.229 |  |
| **CMRF35-like molecule 1 precursor (*S. salar*)** | 0.227 |  |
| **Proteasome activator complex subunit 4-like (*O. niloticus*)** | 0.214 |  |
| **myxovirus resistance 1 (*S. salar*)** | 0.196 |  |
| **FL cytokine receptor precursor (*S. salar*)** | 0.185 |  |
| **metastasis-associated protein MTA1-like (*T. rubripes*)** | 0.184 |  |
| Glyceraldehyde-3-phosphate dehydrogenase (*P. sinensis*) | 0.164 |  |
| dnaJ homolog subfamily C member 11-like (*T. rubripes*) | 0.128 |  |
| **tumor necrosis factor alpha-induced protein 3 (*O. mykiss*)** | 0.0437 |  |
| cholecystokinin receptor type A (*S. salar*) | 0.000 |  |
|  |  |  |
| **common SW1-SW2 (N=24: 12 up, 12 down)** | **SW1 (fc)** | **SW2 (fc)** |
| Ig heavy chain V-III region HPC76 Fragment (*M. musculus*) | 56.8 | 30.5 |
| DNA damage inducible transcript 4 protein (*D. rerio*) | 6.31 | 2.6 |
| T-bet (*O. mykiss*) | 6.21 | 9.16 |
| Barrier-to-autointegration factor A (*X. laevis*) | 3.93 | 1.90 |
| **Nuclear receptor subfamily 1 group D member 2 (*M. musculus*)** | 3.35/5.76 | 2.26/5.46 |
| **Interferon-inducible GTPase 1 (*M. musculus*)** | 3.19 | 2.86 |
| **C-C motif chemokine 19 (*M. musculus*)** | 2.87 | 2.06 |
| **Secretory phospholipase A2 receptor (*P. abelii*)** | 2.61 | 3.10 |
| **Src-like-adapter 2 (*H. sapiens*)** | 2.60 | 2.01 |
| Cholesterol side-chain cleavage enzyme, mitochondrial (*O. mykiss*) | 2.20/2.87 | 2.71/2.81/3.69 |
| MAP kinase-interacting serine/threonine-protein kinase 2 (*H. sapiens*) | 2.17 | 1.89 |
| **NF-kappa-B inhibitor alpha (*S. salar*)** | 1.70 | 0.297 |
| Exocyst complex component 7 (*S. salar*) | 0.398 | 0.235 |
| Hypoxia-inducible factor 1 alpha (*S. salar*) | 0.397 | 0.177 |
| Thymus-specific serine protease (*M. musculus*) | 0.373 | 0.299 |
| **Heat shock cognate 71 kDa protein (*G. gallus*)** | 0.321 | 0.166 |
| **Heat shock cognate 71 kDa protein (*O. latipes*)** | 0.320 | 0.175 |
| Myotubularin-related protein 3 (*R. norvegicus*) | 0.300 | 0.282 |
| **Charged multivesicular body protein 2a (*S. salar*)** | 0.295 | 0.203 |
| **N-acetylmuramoyl-L-alanine amidase (*M. musculus*)** | 0.295 | 0.163 |
| **Heat shock cognate 71 kDa protein (*S. oedipus*)** | 0.276 | 0.210 |
| **Cytokine receptor-like factor 1 (*H. sapiens*)** | 0.203 | 0.189 |
| CD99 antigen precursor (*S. salar*) | 0.128 | 0.257 |
| Saxiphilin (*L. catesbeiana*) | 0.000 | 0.000 |
